# The first definitive Middle Jurassic atoposaurid (Crocodylomorpha, Neosuchia), and a discussion on the genus *T* *heriosuchus*

**DOI:** 10.1111/zoj.12315

**Published:** 2015-09-09

**Authors:** Mark T. Young, Jonathan P. Tennant, Stephen L. Brusatte, Thomas J. Challands, Nicholas C. Fraser, Neil D. L. Clark, Dugald A. Ross

**Affiliations:** ^1^ School of GeoSciences, Grant Institute, The King's BuildingsUniversity of EdinburghJames Hutton RoadEdinburghEH9 3FEUK; ^2^ School of Ocean and Earth Science, National Oceanography CentreUniversity of SouthamptonEuropean WaySouthamptonSO14 3ZHUK; ^3^ Department of Earth Science and EngineeringImperial College LondonLondonSW6 2AZUK; ^4^ National Museums ScotlandChambers StreetEdinburghEH1 1JFUK; ^5^ The HunterianUniversity of GlasgowUniversity AvenueGlasgowG12 8QQUK; ^6^ Staffin Museum6 EllishadderStaffinIsle of SkyeIV51 9JEUK

**Keywords:** Atoposauridae, Bathonian, Crocodyliformes, Scotland, Valtos Sandstone Formation

## Abstract

Atoposaurids were a clade of semiaquatic crocodyliforms known from the Late Jurassic to the latest Cretaceous. Tentative remains from Europe, Morocco, and Madagascar may extend their range into the Middle Jurassic. Here we report the first unambiguous Middle Jurassic (late Bajocian–Bathonian) atoposaurid: an anterior dentary from the Isle of Skye, Scotland, UK. A comprehensive review of atoposaurid specimens demonstrates that this dentary can be referred to *T* *heriosuchus* based on several derived characters, and differs from the five previously recognized species within this genus. Despite several diagnostic features, we conservatively refer it to *T* *heriosuchus* sp., pending the discovery of more complete material. As the oldest known definitively diagnostic atoposaurid, this discovery indicates that the oldest members of this group were small‐bodied, had heterodont dentition, and were most likely widespread components of European faunas. Our review of mandibular and dental features in atoposaurids not only allows us to present a revised diagnosis of *T* *heriosuchus*, but also reveals a great amount of variability within this genus, and indicates that there are currently five valid species that can be differentiated by unique combinations of dental characteristics. This variability can be included in future broad‐scale cladistics analyses of atoposaurids and closely related crocodyliforms, which promise to help untangle the complicated taxonomy and evolutionary history of Atoposauridae.  © 2015 The Authors. Zoological Journal of the Linnean Society published by John Wiley & Sons Ltd on behalf of The Linnean Society of London

## Introduction

Atoposaurids are an extinct clade of terrestrial to semiaquatic crocodyliforms, many of which had a peculiar ‘dwarfed’ body size (Owen, 1879; Joffe, 1967; Buscalioni & Sanz, 1990a; Thies, Windolf & Mudroch, 1997). This clade was first described from Late Jurassic specimens from the lithographic limestones of France and Germany (von Meyer, 1850, 1851). Numerous recent discoveries have identified Atoposauridae as a diverse and specialized group that survived deep into the Cretaceous (Martin, Rabi & Csiki, 2010; Lauprasert *et al*., 2011), and which may have persisted past the Cretaceous/Palaeogene boundary (Stevens *et al*., 2013). Moreover, they were an important component of Late Jurassic to Early Cretaceous terrestrial and semiaquatic ecosystems, with a range of often sympatric species known from across Europe (e.g. Wellnhofer, 1971; Tennant & Mannion, 2014), as well as tentative remains from Asia (Efimov, 1976; Wu, Sues & Brinkman, 1996) Africa (Michard *et al*., 1990), and North America (Cifelli *et al*., 1999a, 1999b; Eaton *et al*., 1999; Fiorillo, 1999).

Most phylogenetic analyses have recovered atoposaurids near the base of Neosuchia (a major lineage including living crocodiles and their closest fossil relatives; Benton & Clark, 1988; Buscalioni & Sanz, 1990b; Salisbury *et al*., 2006; Brochu *et al*., 2009; Pol, Turner & Norell, 2009; Martin *et al*., 2010; Adams, 2013; Sertich & O'Connor, 2014). However, more recent analyses place Atoposauridae as the sister to Paralligatoridae, within Eusuchia, and together comprising the sister to crown group Crocodylia (Turner, 2015; Turner & Pritchard, 2015), suggesting that atoposaurids occupy a hitherto‐unrecognized significant position in the ascent of modern crocodylians.

In spite of the longevity and evolutionary importance of atoposaurids, the origin of this clade is poorly understood owing to a paucity of fossils from the Middle Jurassic, the time span when this clade most likely originated. The earliest known specimens referred to Atoposauridae are isolated tooth crowns from the Bathonian of France and the UK (Evans & Milner, 1994; Kriwet, Rauhut & Gloy, 1997; Knoll *et al*., 2013), as well as undescribed teeth and postcranial remains from the Bathonian of Madagascar (Flynn *et al*., 2006) and Morocco (Haddoumi *et al*., 2015). Many of these specimens have been referred to the atoposaurid genus *Theriosuchus* based on the presence of multiple dental morphotypes, as strongly differentiated (heterodont) dentition characterizes this genus amongst known atoposaurids (Thies *et al*., 1997; Schwarz & Salisbury, 2005; Lauprasert *et al*., 2011). However, as is often the case with isolated teeth, the referral of these specimens to *Theriosuchus* (and Atoposauridae more broadly) is problematic, because it is possible that other crocodylomorphs from the poorly sampled Middle Jurassic also had heterodont dentition or one of the dentition morphotypes usually ascribed to *Theriosuchus*. In order to confirm the presence of atoposaurids in the Middle Jurassic, diagnostic skeletal material is critically needed. If such early and/or basal atoposaurids can be identified, they may help to clarify the taxonomy and internal phylogenetic relationships of Atoposauridae, which remain a major subject of debate (see Owen, 1879; Wellnhofer, 1971; Steel, 1973; Buffetaut, 1982; Clark, 1986; Buscalioni & Sanz, 1988; Brinkmann, 1989, 1992; Wu *et al*., 1996; Schwarz & Salisbury, 2005; and see Martin *et al*., 2010, 2014b; Lauprasert *et al*., 2011; Schwarz‐Wings *et al*., 2011; and Tennant & Mannion, 2014 for recent discussions).

Here we describe a new specimen (the anterior region of a right dentary) from the late Bajocian–Bathonian Valtos Sandstone Formation of the Isle of Skye, Scotland, UK. Detailed comparison with major Jurassic crocodylomorph clades and all known definitive atoposaurid material indicates that this specimen can be assigned to the atoposaurid genus *Theriosuchus* based on a number of newly identified synapomorphies. Although several features appear to be unique to this specimen, we do not erect a new taxon, pending the discovery of further material and future examination of atoposaurid inter‐relationships. As such, this specimen is the oldest known definitive skeletal material of Atoposauridae, and indicates that the oldest atoposaurids were small‐bodied, heterodont taxa. Furthermore, this new specimen and a reassessment of other atoposaurid fossils provide insight into the taxonomy of *Theriosuchus*, a genus whose convoluted taxonomic history has complicated efforts to study atoposaurid phylogeny and evolution.

### Geological information

**Figure 1:**
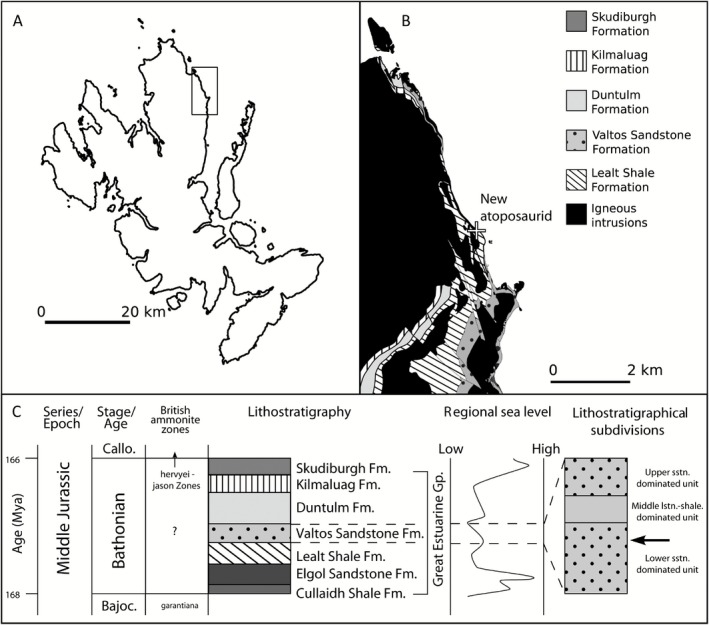
A, geographical; B, geological; and C, stratigraphical setting of the type locality of *T* *heriosuchus* sp., Trotternish Peninsula, Isle of Skye. Ammonite biostratigraphy from Hesselbo, Oates & Jenkyns (1998) and Hesselbo & Coe (2000). The regional sea‐level curve is from Hesselbo & Coe (2000) and Hesselbo (2008). Detailed lithostratigraphical column adapted from Harris (1992). The arrow on the right column approximates the horizon that the type specimen of *Theriosuchus* sp. is believed to have eroded from. Bajoc, Bajocian; Callo, Callovian; Fm., formation; Gp., group; lstn, limestone; Ma, millions of years ago; sstn, sandstone.

The Inner Hebrides of Scotland boasts one of the most complete sequences of Middle Jurassic sedimentary rocks in the world (Morton & Hudson, 1995). These units frequently crop out on the Isle of Skye and often preserve invertebrate and vertebrate fossils (Fig. 1). The Valtos Sandstone Formation is one component of the Great Estuarine Group, and comprises alternating sequences of shelly limestones, mudstones, and sandstones formed during repeated cycles of delta progradation and retrogradation into a marine‐influenced lagoon (Harris, 1992). In his study of the Valtos Sandstone Formation Harris (1992) recognized a relative abundance of fish and reptile remains from his ‘facies 5’, which was characterized by rock‐forming quantities of the bivalve *Neomiodon*. However, the specimen in this study was contained in a silty‐sand matrix similar to ‘facies 1’ of Harris (1992), described as silty, greenish grey bioturbated siltstones with very fine‐grained sand beds. Facies 1 typically directly overlies facies 5 and occurs in the lower sandstone‐dominated units of the Valtos Sandstone Formation between Valtos and Carraig Mhor where the specimen was found.

The Valtos Sandstone can be assigned a late Bajocian–Bathonian age, based on palynological and macrofaunal fossils (Riding *et al*., 1991). Within this age range, the presence of the pollen *Lycopodiacidites baculatus* indicates that the Valtos Sandstone is no younger than early Bathonian, as this taxon does not occur in post‐Bathonian strata in Europe (Riding *et al*., 1991). The Valtos Sandstone preserves a predominantly terrestrial freshwater microflora, as well as rare dinosaur bones and footprints (e.g. Clark & Barco Rodríguez, 1998; Marshall, 2005) and bones of marine reptiles (Brusatte *et al*., 2015).

### Institutional abbreviations

NHMUK, Natural History Museum, London; NMS, National Museums Scotland, Edinburgh; UoB, University of Bucharest, Bucharest.

## Systematic Palaeontology

### Crocodyliformes Benton & Clark, 1988

### Mesoeucrocodylia Whetstone & Whybrow, 1983

### Neosuchia Benton & Clark, 1988

### Atoposauridae Gervais, 1871

### *T* *heriosuchus* Owen, 1878

#### Type species

*Theriosuchus pusillus* Owen, 1879.

#### Etymology

Small crocodile'. *Therio*, from the Greek *therion* (θηρίον), the diminutive of animal/beast, and *suchus* is the Latinized form of the Greek *soukhos* (σοῦχος), the name for one of the species of crocodile living in Ancient Egypt.

#### Paratype

NHMUK PV OR48330, a near‐complete, articulated and three‐dimensionally preserved skull.

#### Lectotype

NHMUK PV OR48216, a near‐complete partially articulated skeleton with skull.

#### Referred species

*Theriosuchus grandinaris* Lauprasert *et al*., 2011; *Theriosuchus guimarotae* Schwarz & Salisbury, 2005; *Theriosuchus ibericus* Brinkmann, 1989; and *Theriosuchus sympiestodon* Martin *et al*., 2010.

#### Generic diagnosis

Atoposaurid crocodyliforms with the following autapomorphic characters: (1) heterodont dentition, with pseudocaniniform, labiolingually compressed, and lanceolate tooth crown morphotypes (labiolingually compressed teeth are absent in *T. guimarotae*); (2) ‘low‐crowned’ teeth present (absent in *T. guimarotae* and *T. grandinaris*; Schwarz & Salisbury, 2005); (3) progressive reduction in alveolus size from the fourth to sixth dentary alveoli; (4) presence of false denticles (serrations created by the superficial enamel ornamentation: pseudoziphodonty, *sensu* Prasad & de Lapparent de Broin, 2002) on the posterior teeth; (5) some of the dentary alveoli form a confluent chain (not visible in the Skye specimen) from dental alveolus D4–D8; (6) enlarged fifth maxillary tooth, typically with a corresponding notch on the dentary (not enlarged in *T. grandinaris*; only moderately enlarged in *T. guimarotae* and *T. pusillus*; unclear in the Skye specimen); (7) maxillary and dentary alveolar size is strongly heterogeneous; (8) dentary external surface is ornamented with heterogeneously spaced pits, and ventrolaterally rugose; and (9) supratemporal fenestrae and fossae proportionally large with respect to primary orbit (supratemporal fossae length can exceed two‐thirds of orbit length in dorsal view).

#### Taxonomic note

It has not been conclusively demonstrated that all currently known *Theriosuchus* species form a monophyletic group (which would be the genus *Theriosuchus*) exclusive of all other atoposaurids, which is reflected in the number of exceptions in the diagnosis presented above. One of the reasons for the high number of exceptions is that most recent taxonomic studies on *Theriosuchus* have not provided an explicit generic diagnosis, typically providing differential species diagnoses that list some craniodental generic characteristics, or detailed discussions (e.g. Brinkmann, 1989, 1992; Martin *et al*., 2010, 2014b; Lauprasert *et al*., 2011).

One exception to this is Salisbury & Naish (2011), who provided a detailed differential diagnosis for *Theriosuchus*, drawing heavily on Schwarz & Salisbury (2005). However, several of the listed characters are present more broadly within Atoposauridae; for example the ‘slit‐like, horizontally oriented and rostrally positioned external nares, separated from each other by the rostral‐most extent of the nasals’ (p. 338), is a feature also found in both *Alligatorellus* (Gervais, 1871) and *Alligatorium meyeri* (Vidal, 1915) (see also Wellnhofer, 1971 and Tennant & Mannion, 2014), as well as the ‘Glen Rose Form'/*Wannchampsus* (Langston, 1974; Rogers, 2003; Adams, 2014). Additional characters, such as a shallow sulcus on the maxilla posterior to the triple junction of the maxilla, premaxilla, and nasal, and features of the posterior mandibular morphology (Schwarz & Salisbury, 2005: 797), cannot be assessed on the Skye specimen.

Furthermore, Martin *et al*. (2010: 847–848) listed some putative generic autapomorphies of *Theriosuchus* in their species diagnosis of *T. sympiestodon*: ‘presence of transversely directed groove on the anterolateral side of the maxilla; longitudinal crest on the frontal; and low‐crowned, labiolingually compressed, pseudoziphodont posterior teeth’. No transverse groove on the maxilla can be seen in the figures of the holotype (*contra* Martin *et al*., 2010, 2014b); moreover, personal examination of this specimen by one of us (J. P. T.) did not detect the presence of this groove. A referred specimen has a depression of some description in this area, at a different orientation to that figure for the holotype specimen (Martin *et al*., 2014b), although we were not able to examine this specimen first hand so we cannot comment on whether it is a groove, bite mark, post‐mortem artefact, or pathological. The presence of a midline frontal crest is likely to be related to ontogeny and the fusion of the frontals, as small individuals of *T. guimarotae* lack this crest (Schwarz & Salisbury, 2005), but the crest is present in adult/large‐bodied *Theriosuchus* specimens (Schwarz & Salisbury, 2005). However, this crest is also present in *Alligatorium meyeri*, *Shamosuchus* (Pol *et al*., 2009), and *Wannchampsus kirpachi* (Adams, 2014; J. P. T., pers. observ.) (a taxon considered to be either an atoposaurid Rogers, 2003, or a paralligatorid Adams, 2014), and therefore is not likely to be diagnostic of *Theriosuchus*.

Many studies have either explicitly stated or implied that variation in tooth morphotypes (heterodonty) is a characteristic feature of *Theriosuchus*. However, not all species of *Theriosuchus* have the same tooth morphology, and other neosuchian crocodyliforms such as bernissartiids have a heterodont dentition (e.g. Sweetman, Pedreira‐Segade & Vidovic, 2015). Therefore, strict heterodonty is not diagnostic of *Theriosuchus*, although more nuanced features, including tooth morphotype combinations, may be diagnostic at the generic and specific level.

Based upon a critical review of all known specimens of *Theriosuchus*, we provide a new diagnosis above. This diagnosis lists nine autapomorphies that are exclusive to *Theriosuchus* and not found in any other atoposaurid, such as *Alligatorellus* or *Alligatorium*. Some of these features are seen in all species of *Theriosuchus*, and others in only a subset (with the exceptions noted). We consider this to be a preliminary diagnosis, because it is based only on comparative anatomy and not a phylogenetic analysis of all atoposaurid specimens. Such an analysis will be necessary to test whether there is a clade uniting all (or some) of the named *Theriosuchus* species and whether the diagnostic characters that we identify here unite this *Theriosuchus* clade and/or subgroups within this clade. An analysis of this breadth is outside of the scope of this paper, and is more properly conducted after the anatomy of new atoposaurid specimens (such as the Skye dentary) have been described in detail. A comprehensive taxonomic, systematic, and phylogenetic revision of Atoposauridae is currently underway by one of us (J. P. T.). The objectives of the present study, therefore, were to determine the taxonomic status of the new Skye specimen based on comparative anatomy and to identify variable characters that may diagnose individual *Theriosuchus* species and unite the *Theriosuchus* species as a clade, which can later be incorporated into phylogenetic analyses.

### *T* *heriosuchus* sp.

#### Referred specimen

**Figure 2:**
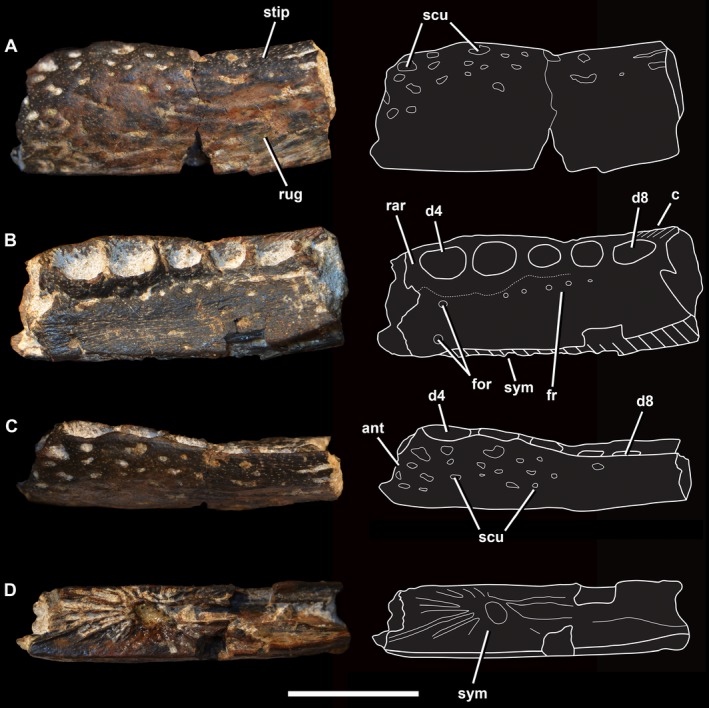
Photographs and line drawings of *T* *heriosuchus* sp. from the late Bajocian–Bathonian Valtos Sandstone Formation of Skye, Scotland. A, ventral view; B, dorsal view; C, lateral view; D, medial view. Abbreviations: ant, anterior edge; c, crenulations; d, dentary tooth positions; for, nutrient foramina; fr, foramen row; rar, raised alveolar rims; rug, rugose texture; scu, sculpting; stip, stippled texture; sym, symphysis. Scale bar = 10 mm.

NMS G. 2014.52.1, an incomplete, three‐dimensionally preserved right dentary. It is broken at both the anterior and posterior ends (Fig. 2).

#### Referred locality

The specimen was collected ex situ in May 2013 by one of us (T. J. C.) from a landslip below Carraig Mhor (British National Grid NG 88715 88607), Trotternish Peninsula, Isle of Skye, Scotland, UK (Fig. 1).

#### Stratigraphical age and horizon

Valtos Sandstone Formation, Great Estuarine Group. Late Bajocian–Bathonian, Middle Jurassic.

#### Differential diagnosis

Atoposaurid crocodyliform within *Theriosuchus* with the following unique combination of characters (putative autapomorphic characters amongst Atoposauridae are indicated by an asterisk *): (1) posterodorsally orientated oblique crenulations (raised ridges) on the medial surface of the D8 and D9 alveoli labial rims (although we cannot entirely preclude these from being taphonomic distortions)*; (2) longitudinally crenulated dorsal dentary surface lingual to the dentary arcade*; (3) distinctive pair of foramina medial to the fourth dentary alveolus, adjacent to the raised D4 alveolar rim and the dorsal margin of the symphysis, respectively* (however, this characteristic varies intraspecifically and requires further study in other *Theriosuchus* specimens; see description below); (4) parallel symphyseal surface to the dentary arcade and external surface*; (5) inferred transition from a pseudocaniniform to labiolingually compressed dental morphotype from D7 to D8 (based on the shape of the alveoli); (6) D4 and D5 almost twice the size of subsequent posterior alveoli; (7) independent dental alveoli separated by interalveolar septae, with no confluent alveoli chain; and (8) no apparent contribution by the splenial to the symphysis*. (Note we cannot entirely discount the possibility that the anterior‐most preserved alveolus is in fact the D3, and not the D4; see below.)

#### Description

The maximum length of the preserved dentary is 26.1 mm, and in dorsal view it has a maximum width of 9.8 mm at the fourth and fifth alveoli (D4–D5), and a minimum width of 9.1 mm at the D6–D7 interalveolar septum (Fig. 2). The dorsoventral height of the symphyseal region in medial view is 6.1 mm, and remains constant along what is preserved of the dentary. The external surface decreases in dorsoventral height, from 5.2 mm adjacent to the D4 alveolus, to 3.7 mm lateral to the D8–D9 alveoli. The medial and lateral surfaces are both straight and parallel in dorsal view, unlike some other members of *Theriosuchus* in which the lateral surface is dorsoventrally convex (Schwarz & Salisbury, 2005; Martin *et al*., 2014b; Young *et al*., 2014b; Table 1).

**Table 1:** Comparison of dentary and alveolar morphologies among atoposaurid specimens

Species	Dentary external surface ornamentation	Anterior dentary lateral margin shape in dorsal view	Symphysis length relative to tooth row	Foramina medial to D4	Vertically festooned alveolar margins	Confluent alveoli	Interalveolar septae	Raised alveolar rims
*Theriosuchus* sp.	Heterogeneously spaced pits	Straight	D7	Yes	Yes	No	Yes	Yes
*Theriosuchus pusillus*	Heterogeneously spaced pits	Laterally convex	D7	Yes	Yes	D3–D4	Yes	Yes
*Theriosuchus guimarotae*	Heterogeneously spaced pits	Laterally convex	D5–D6	No, D2–D3	Yes	D3–D4	Yes	Yes
*Theriosuchus ibericus*	Heterogeneously spaced pits	Straight	D5	No	Yes	No	Yes	Yes
*Theriosuchus grandinaris*	Heterogeneously spaced pits	Straight	D7	?	?	?	?	?
*Theriosuchus sympiestodon*	Heterogeneously spaced pits	Laterally convex	D6	Yes	Yes	D4–D7?	Yes	No
*Atoposaurus jourdani*	?	?	?	?	?	?	?	?
*Atoposaurus oberndorferi*	Smooth	?	?	?	?	?	?	?
*Alligatorellus beaumonti*	Slightly grooved	Straight	?	?	?	?	?	?
*Alligatorium meyeri*	?	?	?	?	?	?	?	?
*Alligatorium franconicum*	?	?	?	?	?	?	?	?
*Alligatorium paintenense*	?	?	?	?	?	?	?	?
*Montsecosuchus depereti*	?	Straight	?	?	?	?	?	?
Putative atoposaurids						?		
*Karatusuchus sharovi*	Smooth	?	?	?	?	?	?	?
*Brillanceausuchus babouriensis*	?	Spatulate	D5	?	?	?	?	?
*Pachycheilosuchus trinquei*	Slightly grooved	Laterally convex	D4	No	No	No	Yes	No

Species	Transitional alveoli (D4+) shape morphology	Alveolar size heterogeneity	Enlarged fifth maxillary tooth	Low‐crowned teeth	Pseudocaniniform tooth crowns	Labiolingually compressed tooth crowns	Lanceolate tooth crowns
*Theriosuchus* sp.	Subcircular to elliptical (D4–D9)	Yes, reduce in size from D4–D8	Inferred from corresponding dentary concavity	?	Inferred based on alveolus shape	Inferred based on alveolus shape	?
*Theriosuchus pusillus*	Subcircular to elliptical (D5–D6)	Yes, reduce in size from D4–D9	Present	Present	Present	Present	Present
*Theriosuchus guimarotae*	Circular to subcircular (D5–D6)	Yes, reduce in size from D4–D7	Present	Absent	Present	Absent	Present
*Theriosuchus ibericus*	Oval to subcircular (D4–D5)	Yes, increase in size from D5–D7	Present	Present	Present	Present	Present
*Theriosuchus grandinaris*	?	Yes, D3 and D4 are enlarged	Absent	Absent	Present	Present	Present
*Theriosuchus sympiestodon*	D4 to D5 becoming more oval	Yes, reduce in size from the D4	Present	?	Present	Present	Present
*Atoposaurus jourdani*	?	?	Absent	Absent	?	Absent	Absent
*Atoposaurus oberndorferi*	?	?	Absent	?	?	?	?
*Alligatorellus beaumonti*	?	?	Absent	Absent	Present	Absent	Absent
*Alligatorium meyeri*	?	?	Absent	Absent	Present	Absent	Absent
*Alligatorium franconicum*	?	?	Absent	?	?	?	?
*Alligatorium paintenense*	?	?	Absent	?	?	?	?
*Montsecosuchus depereti*	?	?	Absent	?	?	?	?
Putative atoposaurids							
*Karatausuchus sharovi*	?	None	Slight	Absent	Present	Absent	Present
*Brillanceausuchus babouriensis*	?	?	Absent	Absent	Present	?	?
*Pachycheilosuchus trinquei*	No, isometric	None	Absent	Absent	Present	?	?

D, dentary alveolus.

The anterior and alveolar external surfaces of the dentary are covered in very small, heterogeneously spaced, subcircular pits, similar to other species of *Theriosuchus* (Owen, 1879; Brinkmann, 1989, 1992; Schwarz & Salisbury, 2005). However, these pits are not as well developed as those in *T. sympiestodon* (Martin *et al*., 2014b). In this pitted region, when larger pits are present they are surrounded by a more abundant stippled pattern on the external surface. Between these large pits the external surface of the bone is generally subtly convex. However, posteriorly and ventrally the external surface becomes more rugose in an anteroposterior direction, with no stippled pattern present.

Five complete dentary alveoli are preserved, with a partial sixth at the posterior end of the bone, which we interpret as being the D4 to D9 alveoli (based upon comparisons with other *Theriosuchus* specimens). We are cautious in this interpretation, however, owing to the fragmentary nature of the specimen. In the Skye specimen, the D4 and D5 alveoli are approximately equal size. In *T. ibericus*, this morphology is unknown, and D4 alveoli are the largest in *T. guimarotae*, *T. pusillus*, and *T. sympiestodon*. In some specimens of *T. pusillus* (e.g. NHMUK PV OR48244), the D3 alveoli are also enlarged, but not to the same degree as the D4. Therefore, we cannot preclude the possibility that the anterior‐most alveolus preserved on the Skye specimen is in fact the D3 alveolus. The preserved alveoli are partially filled with matrix. The maximal anteroposterior lengths of the alveoli are (D4–D8): 3.9, 3.6, 2.7, 2.6, and 3.2 mm. The maximal transverse widths are (D4–D8): 2.6, 2.9, 2.2, 2.1, and 1.8 mm. These show a progressive transformation from a suboval D4–D5, to a subcircular D6–D7, to an anteroposteriorly elongated oval D8, which we interpret as representing a shift from a pseudocaniniform to a labiolingually compressed dental morphology, a feature found only in the heterodont atoposaurid *Theriosuchus*. A similar alveolar shape transition occurs in the Kimmeridgian species *T. guimarotae*, in which a shift from pseudocaniniform dental morphology to a lanceolate morphology is observed, but at the D10 to D11 alveoli (Schwarz & Salisbury, 2005). The transition is also apparent in *T*. *pusillus* but beginning at D5 instead of D7. Furthermore, in *T. pusillus* specimen NHMUK PV OR48244, D10 is occupied by a hypertrophied, lanceolate tooth, with the tooth in D9 representing a transitional morphology between the smaller D5–D8. In *T. sympiestodon*, D4 is clearly the largest, but it is not clear whether D5–D7 occupy a single confluent groove, or are separated by low septae (Martin *et al*., 2014b). There is a clear diastema separating D7 and D8 in *T. sympiestodon*, a feature differentiating this taxon from the Skye specimen. As such, the position of this dental morphology transition, as inferred from alveolar morphology, is a feature that differentiates these species of *Theriosuchus*.

Each tooth in NMS G. 2014.52 occupies an independent alveolus separated by a distinct interalveolar septum. The septum separating the D4 alveolus from the D5 alveolus is thin, whereas all subsequent septa are broader and flatter on the occlusal surface, a condition similar to that of *T. pusillus* between the D3–D4 alveoli (NHMUK PV OR48244). This is unlike the condition apparent in *T. sympiestodon*, in which the D4–D7 alveoli appear to be confluent and the only gap is between the D7 and D8 alveoli (Martin *et al*., 2014b) – however, this is based on a poorly preserved specimen, and we regard this inference as tentative. It is also different from the morphology in *T. pusillus*, which has a confluent D4–D7 alveoli chain (NHMUK PV OR48262; Young *et al*., 2014b), and *T*. *guimarotae*, which has confluent third and fourth alveoli, with the interalveolar bar separating the D4 and D5 alveoli being the broadest of all anterior interalveolar septae (Schwarz & Salisbury, 2005). However, other specimens attributed to this *T. pusillus*, including NHMUK PV OR48244, show that with the exception of D3–D4, the dentary alveoli are separated by septae. In *T. ibericus* all teeth occupy a single continuous dental groove (J. P. T., pers. observ.), a feature that is unlikely to be a result of preservation. Although the validity of *T. ibericus* is questionable (Schwarz & Salisbury, 2005), this feature is enough to distinguish the Skye specimen from this taxon.

The alveoli in NMS G. 2014.52.1 have raised lingual rims, which are more prominent on the D4–D6 alveoli, and vertically festooned labial rims. Although present on *T. guimarotae* (Schwarz & Salisbury, 2005), the development of these rims is much more pronounced on the Skye specimen. Raised lingual rims are not observed on any specimen of *T. pusillus*. On the medial surface of the D8 and D9 alveoli labial rims of NMS G. 2014.52.1 there are oblique crenulations (raised ridges), which extend posterodorsally towards the specimen's posterior break. This feature is not known in any atoposaurid specimen, and therefore we consider it to be diagnostic for the Skye specimen.

Immediately lingual to the dental arcade in NMS G. 2014.52.1 there is a parallel row of foramina extending from the D5 alveolus to at least the D7 alveolus, with at least five foramina present and filled with matrix. This is also seen in *T. guimarotae* and *T. sympiestodon*, but in the latter taxon this row is more medially inset and extends at an angle to the obliquely set symphyseal region. In *T. pusillus* this foramen row either begins adjacent to the D7 alveolus (NHMUK PV OR48262) or adjacent and posteromedial to the D2 alveolus (NHMUK PV OR48244), and runs parallel to the tooth row. The surface that these foramina occupy is flat and longitudinally crenulated in NMS G. 2014.52.1, unlike in *T. guimarotae* and *T. pusillus* in which it is smooth. Furthermore, NMS G. 2014.52.1 has a distinctive pair of lingual foramina medial to D4, with one adjacent to the dorsal symphyseal margin and another adjacent to the raised lingual rim of D4. A single foramen is also present in *T. pusillus*, located more anteromedial to D4 than in NMS G. 2014.52.1. One is also present in *T. guimarotae*, but immediately medial to a possible diastema separating the D2–D3 alveoli. We note that the presence and distribution of these foramina can vary intraspecifically, but to our knowledge there are no atoposaurid specimens that preserve a foramen directly adjacent to the dorsal margin of the symphysis. Therefore, the position of this pair of foramina could be diagnostic for the Skye specimen, but this determination must await further discovery and examination of atoposaurid specimens preserving the dorsal surface of the dentary.

The symphyseal region is parallel to the dental arcade, and terminates lateral to the D7 alveolus, differing from both *T. guimarotae* and *T. pusillus* in which the symphyses are obliquely inclined. Therefore, the parallel orientation of the symphysis is autapomorphic for NMS G. 2014.52.1. Furthermore, the symphysis of the Skye specimen does not appear to receive a contribution from the splenial. However, there is a ‘trough'‐shaped surface posterior to the dentary portion of the symphysis, which might be the sutural surface for the missing splenial. If this is the case, the splenial would extend no further anteriorly than the D6 alveolus, and the total length of the symphysis would have a greater posterior extent, in dorsal view. In *T. pusillus*, the symphysis in both NHMUK PV OR48262 and NHMUK PV OR48244 receives a major contribution from the splenial, extending as far anteriorly as the D3–D4 alveoli in dorsal aspect. In *T. guimarotae*, the splenial contributes to the symphysis anteriorly as far as the D3 alveoli (Schwarz & Salisbury, 2005). The symphyseal suture is comprised of ridges radiating from a central point, resulting in a distinctive ‘starburst’ morphology. These ridges reach the dorsal edge of the medial surface, but terminate before reaching the ventral margin. The ventral region of the medial surface lacking the ridges is instead smooth and unsculptured. This is different to *T. guimarotae* and *T. pusillus*, in which the symphysis occupies the whole medial surface anterior to the D7 alveoli and the D5–D6 alveoli, respectively, and is slightly offset from the posterior medial dental surface, with this area perforated by a distinctive nutrient foramen (*T. pusillus*, NHMUK PV OR48262).

## Discussion

### Taxonomic identification

The specimen NMS G. 2014.52.1 shares diagnostic features with the atoposaurid *Theriosuchus*, and can thus be confidently assigned to this genus (and therefore to Atoposauridae more broadly). Additionally, NMS G. 2014.52.1 does not have the characteristic dentary morphologies of other crocodylomorph clades that were common in the Jurassic, such as thalattosuchians, goniopholidids, pholidosaurids, protosuchians, and sphenosuchians. Here, we review the evidence linking the Skye taxon to Atoposauridae and differentiating it from the other major Jurassic crocodylomorph groups.

###### Exclusion from Thalattosuchia

Thalattosuchians are amongst the most abundant and diverse crocodylomorphs of the Mesozoic. One subgroup of thalattosuchians, Teleosauridae, are the most frequently discovered crocodylomorphs in the Bathonian of the UK. However, the anterior dentary morphologies of the typically longirostrine teleosaurids do not match the Skye specimen. In teleosaurids: (1) the anterior region is spatulate, with the maximal mediolateral width being present at the level of the D3 alveolus; (2) the D3 and D4 alveoli are closely set and are confluent, with the D4 and D5 alveoli separated by a diastema; (3) there are no large foramina medial to the alveoli; (4) the alveolar margins are not vertically festooned; and (5) in dorsal view, the alveolar margin is convex at the D3–D4 region, resulting in those alveoli being positioned dorsal to the D1 and D2 alveoli (e.g. Andrews, 1913; Phizackerley, 1951; Hua, 1999; Lepage *et al*., 2008; Martin & Vincent, 2013; Young *et al*., 2014a). As none of these features is present in the Skye specimen, we can disregard a teleosaurid origin for this specimen.

The other major thalattosuchian clade, Metriorhynchoidea, is known from the Bathonian of Argentina, France and Italy (Eudes‐Deslongchamps, 1867–1869; Mercier, 1933; Gasparini *et al*., 2005; Young *et al*., 2010; Cau & Fanti, 2011). The basal metriorhynchoid *Teleidosaurus calvadosii* has an anterior dentary that is similar to those of teleosaurids, although the transverse expansion at the D3–D4 alveoli is more laterally convex than spatulate (Eudes‐Deslongchamps, 1867–1869). Therefore, NMS G. 2014.52.1, which has a straight lateral margin, can be excluded from *Teleidosaurus*. Basal members of Metriorhynchidae have characteristics that readily preclude NMS G. 2014.52.1 from being a metriorhynchid: (1) festooning along the alveolar margin is either absent or only subtle; (2) no large foramina are present medial to the alveoli; (3) the dentary alveoli lack raised rims; and (4) there is a diastema between the D4 and D5 alveoli (e.g. Andrews, 1913; Lepage *et al*., 2008; Cau & Fanti, 2011; Young *et al*., 2012, 2013). Other Middle Jurassic metriorhynchids, including *Maledictosuchus*, have irregularly spaced alveoli (Parilla‐Bel *et al*., 2013), and can thus be similarly discounted. Therefore, as well as the size differences between metriorhynchids (which are generally quite large) and NMS G. 2014.52.1 (which is very small), there is no character evidence supporting metriorhynchoid affinities for NMS G. 2014.52.1.

###### Exclusion from Goniopholididae

In goniopholidids, the anterior tip of the dentary is laterally convex in dorsal view, resulting in it typically being wider than the region posterior to the D4 alveolus, different to NMS G. 2014.52.1. However, in goniopholidids the ventral and lateral surfaces of the anterior part of the dentary are covered with small, closely spaced pits, similar to the external surface of NMS G. 2014.52.1 (Salisbury *et al*., 1999; Salisbury, 2002; Schwarz, 2002; Smith *et al*., 2010). Although NMS G. 2014.52.1 lacks the D1–D3 region, it is clear from comparing this region of the dentary with those of goniopholidids that it would not have expanded laterally like it does in goniopholidids. Furthermore, goniopholidids are exclusively homodont, with a caniniform dental morphology. This combination of differences means that we can reject NMS G. 2014.52.1 from being a member of Goniopholididae.

###### Exclusion from Pholidosauridae

The anterior end of the dentary is well preserved in many species considered to be pholidosaurids. However, all ‘pholidosaurids’ have: (1) a longirostrine snout morphology; (2) elongate symphyseal sutures (> ten alveoli adjacent); (3) spatulate anterior dentaries, and (4) interalveolar spaces that are variable in size, especially posterior to the spatulate region in which these spaces can be substantially greater in length than the diameter of the adjacent alveoli (e.g. Koken, 1887; Mook, 1933, 1934; Buffetaut & Taquet, 1977; Buffetaut & Ingavat, 1980; de Lapparent de Broin, 2002; Martin *et al*., 2014a). NMS G. 2014.52.1 is most likely not from a nonlongirostrine form, the symphyseal suture is not long (with seven alveoli adjacent), the anterior end of the dentary is not spatulate, and the preserved dentary alveoli are closely set with the interalveolar spaces being less than one quarter of the anteroposterior length of the immediately adjacent alveoli. Therefore, we can disregard NMS G. 2014.52.1 as pertaining to Pholidosauridae.

###### Exclusion from Protosuchia

The holotype of *Protosuchus micmac* is of comparable size and has a similar dentary external ornamentation pattern as NMS G. 2014.52.1 (Sues *et al*., 1996). However, *Protosuchus* can be readily differentiated based on its distinctive dorsolateral expansion at the D3–D4 alveoli, a characteristic also seen in other protosuchians (e.g. *Sichuanosuchus shuhanensis*; Wu, Sues & Dong, 1997). *Sichuanosuchus huidongensis* has a spade‐shaped symphysis that is broader than long (Peng, 1996), unlike NMS G. 2014.52.1. Additionally, the symphysis extends further posteriorly to the D8 alveolus in protosuchians (e.g. *Shantungosuchus*; Wu, Brinkmann & Lu, 1994). The D3–D4 alveoli become distorted into laterally inclined ellipses to accommodate the fully caniniform C1 and C2 teeth in protosuchians (Sues *et al*., 1996). This is clearly not the case in NMS G. 2014.52.1, in spite of the absence of the D3 alveolus. Finally, protosuchians exhibit a pattern of alveoli reduction in the D5–D6 alveoli followed by the size increase from the D7 alveolus onwards, also distinct from NMS G. 2014.52.1. Therefore, based on this combination of differences, we can exclude NMS G. 2014.52.1 from Protosuchia.

###### Exclusion from Sphenosuchia

NMS G. 2014.52.1 is also different to ‘sphenosuchians’ (i.e. noncrocodyliform crocodylomorphs), such as *Macelognathus vagans*, which have dorsoventrally flattened dentaries with isometric alveoli, each separated by a bony septum (Göhlich *et al*., 2005). Additionally, sphenosuchians, such as *Dibothrosuchus*, *Junggarsuchus*, and *Terrestrisuchus* have smooth dentary external surfaces (Crush, 1984; Wu & Chatterjee, 1993; Clark *et al*., 2004), unlike the well‐ornamented external surfaces of NMS G. 2014.52.1. Based on these differences, we can exclude NMS G. 2014.52.1 from Sphenosuchia.

###### Inclusion within Atoposauridae

Atoposaurids share with NMS G. 2014.52.1: (1) a nonspatulate anterior dentary (an ambiguous synapomorphy of the group); (2) a notable pair of foramina medial to the alveoli, the position of which is variable amongst taxa and possibly individuals; (3) raised lingual alveolar rims adjacent to the D4–D6 alveoli; (4) vertically festooned alveolar margins; (5) a symphyseal suture of moderate length (five to seven alveoli adjacent; note that this is taking into account the possible exclusion of the splenial from the symphysis); (6) a strongly ornamented external dentary surface, comprising both subcircular pits and anteroposterior grooves; and (7) inferred dental heterodonty and accompanying morphology of the dental alveoli, diagnostic of the atoposaurid *Theriosuchus* (e.g. Owen, 1879, Brinkmann, 1992; Schwarz & Salisbury, 2005; Lauprasert *et al*., 2011; Martin *et al*., 2014b). Furthermore, the size of the Skye specimen is consistent with a crocodyliform of small size, often considered to be diagnostic of atoposaurids.

Amongst known atoposaurids, very few specimens preserve the anterior dentary in three dimensions, and in particular the alveolar region. *Alligatorellus*, *Alligatorium*, *Atoposaurus*, and *Montsecosuchus*, as well as the putative atoposaurids *Brillanceausuchus* and *Karatausuchus*, all preserve skulls, but are dorsally or dorsolaterally flattened and do not preserve the dentary and dental arcade in a manner that facilitates comparison with NMS G. 2014.52.1 (Wellnhofer, 1971; Efimov, 1976; Buscalioni & Sanz, 1990a; Michard *et al*., 1990; Storrs & Efimov, 2000; Tennant & Mannion, 2014). Amongst the multispecific genus *Theriosuchus*, *T. grandinaris* preserves the anterior‐most dentary in ventral view, and it is also three‐dimensionally preserved in *T. guimarotae* (Schwarz & Salisbury, 2005), *T. ibericus* (Brinkmann, 1989, 1992), *T. pusillus* (Owen, 1879; Salisbury, 2002; Young *et al*., 2014b), and material ascribed to *T. sympiestodon* (Martin *et al*., 2014b). This relative rarity of preservation means that, to date, no dentary synapomorphies have ever been consistently identified amongst atoposaurids (e.g. Wellnhofer, 1971; Buscalioni & Sanz, 1990b; but see Schwarz & Salisbury, 2005), although within *Theriosuchus* there are numerous characteristics worth noting, which we list in our preliminary diagnosis and outline here.

Within Atoposauridae, NMS G. 2014.52.1 has one definitive alveolar characteristic that links it to some species of *Theriosuchus*: the posterior symphyseal dentary alveoli shift from being subcircular to anteroposteriorly elongate in shape (Owen, 1879; Lauprasert *et al*., 2011; Martin *et al*., 2014b; Young *et al*., 2014b). As shown by the well‐preserved dentary and dentition of *T. pusillus* (NHMUK PV OR48262 and NHMUK PV OR48244), this change in alveolar shape corresponds to a transitional change in tooth morphology across the dentition, from teeth with ‘pseudocaniniform’ morphology anteriorly to those with a labiolingually compressed form posteriorly. The two end‐members of this transitional sequence are the tooth morphotypes that define the consistently heterodont *Theriosuchus*, and which are commonly used to refer isolated teeth to Atoposauridae. This transitional alveolar morphology is a unique characteristic for *T. pusillus* (Lauprasert *et al*., 2011; Young *et al*., 2014b) and *T. sympiestodon* (Martin *et al*., 2014b) amongst definitive atoposaurids. All other species of *Theriosuchus* have no transitional morphology, either having consistently (sub)circular alveoli (*T. guimarotae*), or all teeth occupying a single dental groove (*T. ibericus*), or being unknown (*T. grandinaris*). All other known atoposaurid species in other genera appear to have a pseudocaniniform, homodont dentition (with the exclusion of *Atoposaurus* and *Montsecosuchus*, for which the dentition is unknown; Wellnhofer, 1971; Buscalioni & Sanz, 1990a; Tennant & Mannion, 2014).

An identical pattern of progressive reduction in alveolus size posteriorly to the D4 alveolus in *T. pusillus*, as well as the presence of a nutrient foramen medially to the D4 alveolus (NHMUK PV OR48262; foramen absent in NHMUK PV OR 48244), provide evidence for association of NMS G. 2014.52.1 with *T. pusillus*. NMS G. 2014.52.1 is nearly identical to these specimens in this morphology, except for the constant presence of interalveolar septae from at least D4–D8 (including either side of these terminally preserved alveoli) instead of a confluent ‘chain’ of alveoli, the anteroposterior extent of which may be autapomorphic for different species of *Theriosuchus*. Septae may be present in *T. pusillus*, but no farther posteriorly than subsequent to the D5 alveolus (Lauprasert *et al*., 2011), and when the septae are present, the spacing between alveoli in *T. pusillus* is closer than that for the Skye specimen. NMS G. 2014.52.1 shares additional features with various species of *Theriosuchus*, including seven teeth adjacent to the mandibular symphysis, alveoli that are heterogeneous in size (as well as shape, see above), and external dentary surfaces sculpted with heterogeneously spaced pits (Table 1).

**Figure 3:**
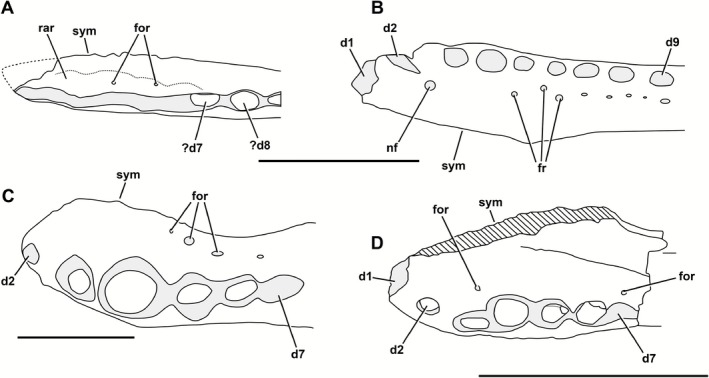
Line drawings of the dentaries of *T* *heriosuchus* species in dorsal aspect. A, *T* *heriosuchus ibericus*; B, *T* *heriosuchus guimarotae*; C, *T* *heriosuchus sympiestodon*; D, *T* *heriosuchus pusillus*. Abbreviations: d, dentary tooth positions; for, nutrient foramina; fr, foramen row; nf, nutrient foramen; rar, raised alveolar rims; sym, symphysis. Scale bars = 10 mm.

Although NMS G. 2014.52.1 shares derived characteristics with *Theriosuchus*, it can be excluded from all known species of this genus based on a combination of distinctive morphological differences (Table 1; Fig. 3). First, as outlined above, NMS G. 2014.52.1 does not have the confluent chain of alveoli seen in some specimens of the Kimmeridgian to Berriasian *T*. *pusillus* (posteriorly from the D4 alveoli in NHMUK PV OR48262). Furthermore, NMS G. 2014.52.1 is distinct from *T. pusillus* in having a parallel symphysis and dentary arcade, the presence of a dual foramen pair on the occlusal surface (although this may vary within species), and a proportionally larger D5 alveolus (compared with NHMUK PV OR 48244 and NHMUK PV OR48262). Second, NMS G. 2014.52.1 cannot be referred to the Kimmeridgian species *T. guimarotae* (Schwarz & Salisbury, 2005) because of the lack of labiolingually compressed dentition in the latter. Additionally, the symphysis in *T. guimarotae* is oblique to the dental arcade, not parallel as in NMS G. 2014.52.1. NMS G. 2014.52.1 is also clearly different from the Barremian *T. ibericus* and the Maastrichtian *T. sympiestodon*, as these two species share a hypertrophied fifth maxillary tooth (Brinkmann, 1989, 1992; Martin *et al*., 2010, 2014b), which creates a notch in the associated portion of the dentary, whereas in NMS G. 2014.52.1 there is a shallow dorsal concavity. Note that we disagree with Martin *et al*. (2010, 2014b) on the hypertrophy of the M4 in *T. sympiestodon*, as we consider the large tooth in this specimen to be the M5 tooth, as the structure that they label as a foramen is more probably the M1 alveolus (J. P. T., pers. observ.). Additionally, the D4 and D5 alveoli are much more equal in size in NMS G. 2014.52.1 relative to the condition in *T*. *sympiestodon* (in which the D4 is about four times the size of D5), and in NMS G. 2014.52.1 the D5 alveolus retains its subcircular shape whereas in *T. sympiestodon* it is much more labiolingually compressed. Furthermore, the lateral margin of the dentary in *T. sympiestodon* is laterally convex in dorsal aspect at the D4–D5 alveoli, whereas in NMS G. 2014.52.1 it is straight. Finally, additional morphological features differentiate NMS G. 2014.52.1 from *T. ibericus*, in which the dentary of the latter is strongly mediolaterally compressed, with all anterior alveoli occupying a single, continuous dental groove.

NMS G. 2014.52.1 shares with *T. grandinaris* (Early Cretaceous of Thailand) a long mandibular symphysis, which has seven alveoli adjacent. Lauprasert *et al*. (2011) considered the long symphysis to be an autapomorphy of *T. grandinaris*. However, it is also this length in *T. pusillus*, and therefore may unite a subgroup within *Theriosuchus*. The dentary arcade is not known in *T. grandinaris* owing to the fusion of the upper and lower jaws during preservation. Nonetheless, NMS G. 2014.52.1 can be excluded from *T. grandinaris* based on differences in alveolar size heterogeneity, the inferred presence of a larger fifth maxillary tooth, and parallel symphysis and dental arcade in the Skye taxon (Lauprasert *et al*., 2011).

Therefore, in summary, the Skye specimen can be excluded from most Mesozoic crocodylomorph groups but shares numerous features with atoposaurids and so can be confidently assigned to the multispecific atoposaurid taxon *Theriosuchus* based on apomorphies.

### Evolutionary and palaeobiogeographical importance of *T* *heriosuchus* sp.

The new crocodyliform specimen from Skye represents the oldest definitive specimen of *Theriosuchus*, and the oldest clearly diagnostic member of Atoposauridae. This provides several important insights into the evolution and distribution of this clade, which would evolve into a speciose group that persisted for around 100 000 000 years and became widely distributed across the globe. First, the Skye specimen indicates that atoposaurids were present by the Bajocian to Bathonian, and had evolved their characteristic small body size and heterodont dentition by this time. Although the morphology of the oldest known specimens does not necessarily indicate the plesiomorphic conditions of a clade, the antiquity of the Skye specimen and its general similarity to later atoposaurids suggests that small size and heterodont dentition were ancestral features of this group. This hypothesis can be tested through character optimization on a comprehensive phylogeny of Atoposauridae, which is currently in development by JPT.

The presence of a diagnostic skeletal fossil of Atoposauridae in the Middle Jurassic lends support to the atoposaurid identification of isolated heterodont teeth from the Bathonian of Madagascar, France, and the UK (Evans & Milner, 1994; Kriwet *et al*., 1997; Flynn *et al*., 2006; Knoll *et al*., 2013). Previously, their atoposaurid identity was based on similarity to the heterodont dentition of *Theriosuchus*, but without diagnostic skeletal fossils of atoposaurids from this time it was possible that these teeth belonged to a different group of heterodont crocodylomorphs (or other archosaurs). Although that is still a possibility (as is always the case with isolated teeth), the new specimen from Skye is definitive evidence that atoposaurids were present during the Bathonian, and as such are the primary candidate for the identity of these distinctive teeth. Further examination of associated body fossils, such as those preliminarily identified by Flynn *et al*. (2006), as well as recently identified small indeterminate neosuchian fossils from the Bathonian Kilmaluag Formation of the Isle of Skye that potentially could belong to atoposaurids or their close relatives (Wills, Barrett & Walker, 2014), should provide much key insight into the early radiation of atoposaurids.

The occurrence of *Theriosuchus* sp. provides a new datum point that, in concert with the tooth record, indicates that atoposaurids were fairly geographically widespread during the very earliest part of their recorded history. This may seem at odds with the limited fossil record of this group during this time, but their small size may explain their rarity. This should stimulate more detailed examination of the biogeography and place of origin of Atoposauridae. It is always tempting to hypothesize that a group originated where its earliest fossils were found, but the patchy Middle Jurassic record of atoposaurids renders such speculation unwise at this point. In the future, however, cladistic biogeographical analysis could be applied to a species‐level phylogeny of Atoposauridae to better understand their distribution and biogeographical evolution over time.

Here we have identified numerous dentary and dentition characteristics that vary in *Theriosuchus*, and possibly more broadly amongst atoposaurids. Coupled with their distinct heterodonty, these morphological differences may indicate that dietary specialization, or character displacement relating to prey choice, was a major driver of early atoposaurid evolution. This variation is also likely to be phylogenetically informative, and may help to elucidate the poorly understood evolutionary relationships within Atoposauridae. To our knowledge, the variation in dentition and dentary morphologies described herein has never been included in a comprehensive analysis of atoposaurid relationships. Conducting a phylogenetic analysis is out of the scope of this paper, although it is in progress by J. P. T., along with a systematic re‐evaluation of the other *Theriosuchus* species. The variable dentary and dentition characters that we have outlined here will be incorporated into this analysis.

### Status of *T* *heriosuchus*

Alongside the description of *Theriosuchus* sp. here, the genus *Theriosuchus* contains five different species: *T. guimarotae* from the Kimmeridgian of Portugal (Schwarz & Salisbury, 2005); *T. pusillus* from the Kimmeridgian to Berriasian of the UK (Owen, 1879); *T. ibericus* from the Barremian of Spain (Brinkmann, 1989, 1992); *T. grandinaris* from the Berriasian to early Barremian of Thailand (Lauprasert *et al*., 2011); and *T. sympiestodon* from the Maastrichtian of Romania (Martin *et al*., 2010, 2014b). The status of these species is in some flux, with *T. ibericus* being of questionable validity (Schwarz & Salisbury, 2005; Lauprasert *et al*., 2011).

**Figure 4:**
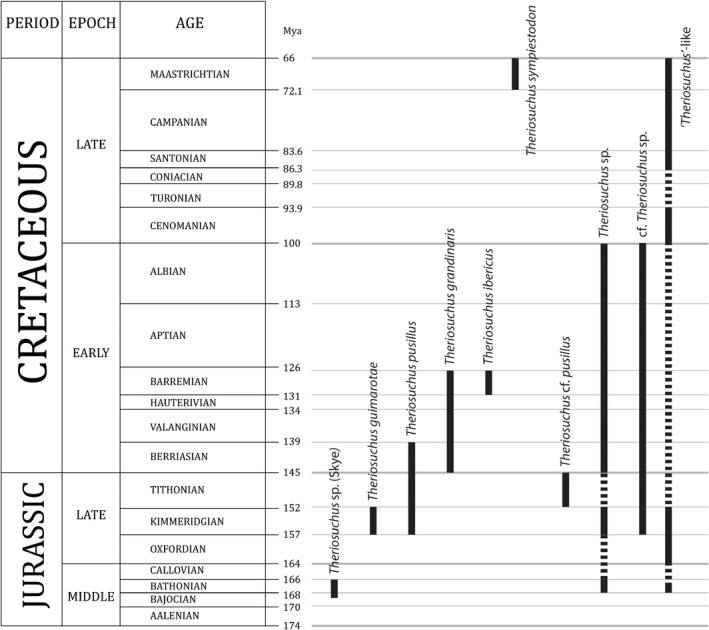
Chronostratigraphical chart of the *T* *heriosuchus* species, cf. *T* *heriosuchus* specimens, and *T* *heriosuchus*‐like specimens. See the Discussion for the relevant references.

**Figure 5:**
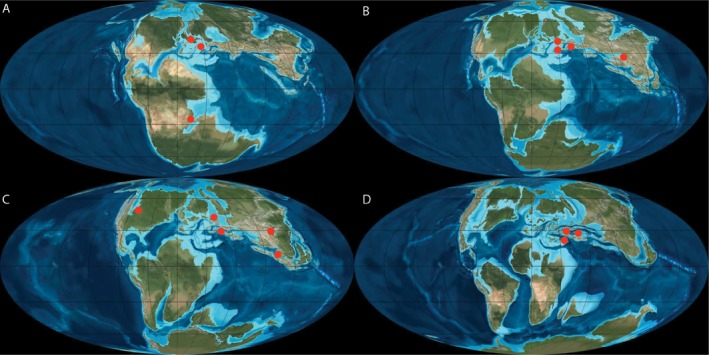
Map of *T* *heriosuchus* and *T* *heriosuchus*‐like specimens from the (A) Middle Jurassic, (B) Upper Jurassic, (C) Lower Cretaceous, and (D) Upper Cretaceous. See the Discussion for the relevant references. Palaeomaps are modified version of high‐resolution versions kindly provided by Ron Blakey (http://cpgeosystems.com/).

Alongside these remains, a multitude of material from the Jurassic and the Cretaceous has been attributed to *Theriosuchus*, or described as having a *Theriosuchus*‐like morphology, with differing degrees of confidence (Figs 4 and 5). These include: atoposaurid teeth similar to those of *Theriosuchus* from the early Bathonian of southern France (Kriwet *et al*., 1997); a fragmentary atoposaurid tooth from the middle Bathonian of southern France (Knoll *et al*., 2013); indeterminate teeth and possible but not clearly diagnostic cranial and osteoderm remains from the Bathonian of Madagascar (Flynn *et al*., 2006); an indeterminate but *Theriosuchus*‐like set of teeth from the Oxfordian of north‐west China, assigned to Mesoeucrocodylia indet. (Wings *et al*., 2010); *Theriosuchus*‐like teeth assigned to a dwarf mesosuchian from the Kimmeridgian of northern Germany (Thies & Broschinski, 2001); another specimen comprising the anterior part of crushed skeleton from the Kimmeridgian of northern Germany tentatively referred to *Theriosuchus pusillus* by Karl *et al*., 2006); and cf. *Theriosuchus* sp. from north‐western Germany (Thies *et al*., 1997). Moreover, isolated teeth from the Tithonian of northern France have been referred to cf. *Theriosuchus* sp. (Cuny *et al*., 1991), whereas isolated teeth from the Tithonian of western France have been referred to *Theriosuchus* cf. *pusillus* (Vullo *et al*., 2014). Other Cretaceous specimens include: *Theriosuchus* sp., tentatively referred from the Early Cretaceous of Utah (USA; Fiorillo, 1999); *Theriosuchus* sp. based on a tooth from the Berriasian of south‐west France (Pouech, Mazin & Billon‐Bruyat, 2006; Pouech *et al*., 2014); *Theriosuchus* sp. from the Berriasian of Sweden and Denmark (Schwarz‐Wings, Rees & Lindgren, 2009); a skull fragment described as *Theriosuchus* sp. from the Wealden of the Isle of Wight (Berriasian–Barremian; Buffetaut, 1983); a range of material ascribed to cf. *Theriosuchus* sp., *Theriosuchus* sp., and *Theriosuchus* cf. *pusillus* from the Early Cretaceous of Thailand (Cuny *et al*., 2010) and cf. *Theriosuchus* sp. from Inner Mongolia, China (Wu *et al*., 1996). In the Late Cretaceous, there are *Theriosuchus*‐like teeth from the Cenomanian of south‐western France, ascribed to Atoposauridae (Vullo & Néraudeau, 2008); *Theriosuchus*‐like teeth from the Santonian of western Hungary, conservatively referred to Mesoeucrocodylia indet. (Ösi *et al*., 2012); and a *Theriosuchus*‐like tooth from the Campanian–Maastrichtian of Portugal (Galton, 1996), although this tooth may be more comparable to *Bernissartia* (Lauprasert *et al*., 2011).

These specimens, if all from the same lineage, would undoubtedly make *Theriosuchus* one of the longest‐lived and most successful crocodylomorphs of the Mesozoic. However, most of these taxonomic identifications are based on teeth, which makes precise referrals to *Theriosuchus* based on apomorphies difficult, as well as creating issues in constructing species‐level diagnoses and deciphering the relationships of *Theriosuchus*. At the very least, most authors agree that *Theriosuchus* (and the various specimens assigned to the lineage) comprise a subgroup of Atoposauridae, and recent phylogenetic analyses have demonstrated the atoposaurid affinities of *Theriosuchus* with strong support (e.g. Martin *et al*., 2010). Some authors take a different view, however, with Buffetaut (1982, 1983) and Kälin (1955) considering *Theriosuchus* to be distinct enough to form a separate family from Atoposauridae, although this idea has not gained much traction in recent years. However, the monophyly of all known *Theriosuchus* species has yet to be tested.

The heterodont dentition of *Theriosuchus* has been considered diagnostic for the genus since first described by Owen (1879), with four distinct morphotypes present: (1) pseudocaniniform tooth crowns, with lingual apicobasally aligned enamel ridges, typically found in the anterior‐most regions of the premaxillae and dentaries; (2) lanceolate‐shaped tooth crown crowns with lingual striae, which are present in the middle to posterior sections of the jaws and have a constriction at the crown–root junction; (3) a low‐crowned morphotype present only in *T. pusillus, T. ibericus*, and *T. sympiestodon*; and (4) broad and labiolingually compressed teeth with striations on the labial and lingual faces, found in *T. ibericus* and *T. pusillus*, and inferred for the dentary of the Skye specimen based on the shape of the alveoli (Owen, 1879; Brinkmann, 1992; Thies *et al*., 1997; Salisbury, 2002; Schwarz & Salisbury, 2005; Schwarz‐Wings *et al*., 2009). *Theriosuchus* sp. would have had at least two of these morphotypes, as inferred from alveolus shape (the pseudocaniniform morphotype in anterior alveoli, and either the lanceolate or labiolingually compressed morphotype in more posterior dentary alveoli), representing a transitional heterodont morphology that strongly unites the specimen with other *Theriosuchus* specimens. We conclude here, based on our comparative study of all *Theriosuchus* material (Table 1), that this form of heterodonty is a diagnostic feature of *Theriosuchus*, and that isolated teeth fitting one or more of these morphotypes are likely to be referable to *Theriosuchus*.

The new mandibulodental characteristics described here indicate that a unique dental morphology is present in each previously recognized species of *Theriosuchus* (Table 1), reaffirming their validity. We therefore tentatively accept the presence of five distinct known *Theriosuchus* species. Combinations of these dental characteristics may identify more inclusive subclades within *Theriosuchus*, or show that some species of *Theriosuchus* may be more closely related to other atoposaurid genera (in which case, this would suggest that the genus *Theriosuchus* should be restricted to the type species and those other taxa most closely related). However, testing of this and the taxonomic validity of these species will require a detailed phylogenetic analysis and comparative anatomical study amongst all currently known species.

## Conclusions

Herein we have described *Theriosuchus* sp., a new specimen of atoposaurid crocodyliform from the Bathonian of the Isle of Skye, Scotland. The species is referred to Atoposauridae, and more specifically to the genus *Theriosuchus*, based on derived characters, including a new suite of dentary and dental synapomorphies that we have outlined above. This occurrence of *Theriosuchus* sp. demonstrates that both Atoposauridae and the genus *Theriosuchus* evolved by the Middle Jurassic (*c.* 168 Mya), lending further support to previous identifications of Bathonian atoposaurids based on isolated teeth. The Middle Jurassic record of atoposaurids demonstrates that this clade achieved a widespread distribution early in its history, and the size and morphology of the new Skye material indicate that small body size and complex heterodont dentition, both characteristic features of atoposaurids, evolved early and may have been ancestral features for the group. The high variability in atoposaurid dental and dentary characteristics identified and synthesized here show that atoposaurid lower jaw and dental evolution is more complex than previously realised. Until now, such variation has been largely overlooked, but promises great utility in atoposaurid taxonomy, systematics, and phylogeny. Inclusion of these characters within a comprehensive phylogenetic analysis, including all species referred to *Theriosuchus* as well as other established and putative atoposaurids, will provide insight into the pattern of evolution of dental characteristics within Atoposauridae, the internal status of *Theriosuchus* and its relationships to other atoposaurids, and the evolution of characteristic features and biogeographical distribution in this bizarre and widespread Mesozoic crocodylomorph group.
